# Supplementary material for: Preclinical characterization of an intravenous coronavirus 3CL protease inhibitor for the potential treatment of COVID19
Source: Nat Commun. 2021 Oct 18;12:6055. doi: 10.1038/s41467-021-26239-2 (PMC8523698; doi:10.1038/s41467-021-26239-2)
Supplement: Supplementary file 1 — Supplementary Information [file 41467_2021_26239_MOESM1_ESM.pdf]

Title: **Preclinical Characterization of an Intravenous Coronavirus 3CL Protease Inhibitor for the Potential Treatment of COVID19**

Authors: Britton Boras[1,†], Rhys M. Jones[1,†,*], Brandon J. Anson[2], Dan Arenson[3], Lisa Aschenbrenner[3], Malina A. Bakowski[4], Nathan Beutler[5], Joseph Binder[1], Emily Chen[4], Heather Eng[3], Holly Hammond[6], Jennifer Hammond[7], Robert E. Haupt[6], Robert Hoffman[1], Eugene P. Kadar[3], Rob Kania[1], Emi Kimoto[3], Melanie G. Kirkpatrick[4], Lorraine Lanyon[3], Emma K. Lendy[8], Jonathan R. Lillis[9], James Logue[6], Suman A. Luthra[10], Chunlong Ma[11], Stephen W. Mason[12,3], Marisa E. McGrath[6], Stephen Noell[3], R. Scott Obach[3], Matthew N. O' Brien[13], Rebecca O'Connor[3], Kevin Ogilvie[3], Dafydd Owen[10], Martin Pettersson[10], Matthew R Reese[3], Thomas F. Rogers[5,14], Romel Rosales[15,16], Michelle I. Rossulek[10], Jean G. Sathish[12], Norimitsu Shirai[3], Claire Steppan[3], Martyn Ticehurst[9], Lawrence W. Updyke[10], Stuart Weston[6], Yuao Zhu[12], Kris M. White[15,16], Adolfo García-Sastre[15,16], Jun Wang[11], Arnab K. Chatterjee[4], Andrew D. Mesecar[2,8], Matthew B. Frieman[6], Annaliesa S. Anderson[12], Charlotte Allerton[10]

Affiliations:

[1]Worldwide Research and Development, Pfizer Inc., La Jolla, CA 92121,

[2]Department of Biological Sciences, Purdue University, West Lafayette, IN, 47907 USA.
[3]Worldwide Research and Development, Pfizer Inc., Groton, CT 06340 USA

[4]Calibr, a division of The Scripps Research Institute, La Jolla, CA 92037 USA

[5]Department of Immunology and Microbiology, The Scripps Research Institute, La Jolla, CA 92037
[6]Department of Microbiology and Immunology University of Maryland School of Medicine, Baltimore, MD 21201
[7]Worldwide Research and Development, Pfizer Inc., Collegeville, PA 19426, USA

[8]Department of Biochemistry, Purdue University, West Lafayette, IN, 47907 USA
[9]Worldwide Research and Development, Pfizer Inc., Sandwich, CT13 9ND, UK
[10]Worldwide Research and Development, Pfizer Inc., Cambridge, MA 02139 USA

[11]Department of Pharmacology and Toxicology, College of Pharmacy, University of Arizona, Tucson, AZ, 85721
[12]Worldwide Research and Development, Pfizer Inc., Pearl River, NY 10965 USA
[13]Worldwide Research and Development, Pfizer Inc., Lake Forest, IL 60045 USA

[14]UC San Diego Division of Infectious Diseases and Global Public Health, UC San Diego School of Medicine, La Jolla, CA 92093.
[15]Department of Microbiology, Icahn School of Medicine at Mount Sinai, New York, NY, USA
[16]Global Health and Emerging Pathogens Institute, Icahn School of Medicine at Mount Sinai, New York, NY, USA

† These authors contributed equally to this work.

* To whom correspondence should be addressed. Email: rhys.jones@pfizer.com

This Supplement includes:

Figures S1-S3
Tables S1-S12
References (1-7)

Physiologically-based pharmacokinetic (PBPK) modeling of PF-00835231

A commercially available dynamic PBPK model, Simcyp population-based simulator (version 18.2; Certara UK Limited, Simcyp Division, Sheffield, United Kingdom), was used in the present study[1]. Physicochemical and pharmacokinetic parameters of PF-00835231 for the PBPK models are summarized in Table S11. Since PF-07304814 (prodrug) was predicted to be converted to PF-00835231 rapidly and extensively *in vivo*, the simulation was performed assuming an intravenous infusion of PF-00835231 with the conversion efficiency of 75% from PF-07304814, which was predicted from animal data. For the prediction of DDIs of PF-00835231 with itraconazole, the vendor-verified compound files in Simcyp library were used, i.e., itraconazole (sv-itraconazole_fed capsule) with competitive K_i = 0.0013 µM and itraconazole metabolite (sv-OH-itraconazole) with competitive K_i = 0.0023 µM.

Simulation of clinical trials was performed with a virtual population of healthy volunteers in 10 trials of 10 subjects (total 100 subjects), each aged 20 to 50 years with a female/male ratio of 0.5, whose CYP3A4 degradation rate constant (k_{deg}) was 0.019 h^{-1} in liver and 0.030 h^{-1} in intestine. The output sampling interval in Simcyp simulation toolbox was set to 0.2 hours in all simulations. To predict DDIs of PF-00835231 with itraconazole, PF-00835231 at 320mg/day (equivalent to PF-00835231 formed following PF-07304814 ~500mg/day taking into account conversion and molecular weight differences) was administered IV for 10 days (days 5 to 15) to a virtual population with and without 15-day repeated oral administration of itraconazole 200mg once daily (days 1 to 15). Pharmacokinetic parameters such as maximal plasma concentration (C_{max}), area under the plasma concentration time-curve from time zero to 24 hours post dose (AUC) and the ratios of C_{max} (C_{max}R) and AUC (AUCR) in treatment groups relative to control groups were obtained from Simcyp outputs.

The model-predicted C_{max}R and AUCR for PF-00835231 were ~2x at the daily dose of 320mg/day (corresponding to ~500mg/day PF-07304814) (Table S12).

C_{eff} projection of protease inhibitor to the clinic

The inhibitory quotient (IQ) has been a useful metric for translating preclinical antiviral potencies to the clinic across a number of viral diseases as indicated in the FDA guidance[2]. IQ is defined as the human $C_{min,u}$ unbound concentration divided by the *in vitro* unbound (serum adjusted) $EC_{50,u}$ value in the antiviral assay (equation 13).

$$IQ = \frac{C_{min,u}}{EC_{50,u}} \quad (13)$$

Some antiviral therapies have shown significant benefit with IQ close to 1[3]; however, rapidly controlling viral replication frequently requires maintaining an exposure at least 10x higher than *in vitro* EC_{50}[4]. Clinically approved protease inhibitors have effectively decreased viral loads when dosed at IQ values from 1-100, when protein binding and site of action exposure are taken into account[4]. Importantly, antivirals in general and, specifically, protease inhibitors can potentially lead to increased mutations and additional drug resistance when dosed at an IQ less than 1[5].

How high an IQ value is required depends on the steepness of the dose response curve. The hill coefficient (m), and the EC_{50} are related to the *in vitro* antiviral activity at a range of concentrations (C) by equation 14:

$$\text{in vitro antiviral activity} = 100 * \frac{\mathrm{C}^{\mathrm{m}}}{\mathrm{EC}_{50}^{\mathrm{m}} + \mathrm{C}^{\mathrm{m}}} \quad (14)$$

PF-00835231 shows a high hill coefficient (m=3) across a range of *in vitro* antiviral assays, like those of clinical protease inhibitors targeting HIV and HCV[6,7]. There is only a 2- to 3-fold difference between the antiviral EC_{50} and EC_{90} concentrations (Fig. 2), rather than the typical 9-fold difference for antiviral agents with hill coefficients of 1. Therefore, relatively small ratios of exposure to EC_{50} values (3-10) are related to near complete viral suppression.

References

1. Jamei, M. *et al.* The Simcyp Population-based ADME Simulator. *Expert Opin. Drug Metab. Toxicol.* **5**, 211–223 (2009).
2. *Guidance for Industry Antiviral Product Development - Conducting and Submitting Virology Studies to the Agency*. https://www.fda.gov/media/71223/download (2006).
3. Mo, H. *et al.* Estimation of inhibitory quotient using a comparative equilibrium dialysis assay for prediction of viral response to hepatitis C virus inhibitors. *J. Viral Hepat.* **18**, 338–348 (2011).
4. Reddy, M. B. *et al.* Pharmacokinetic/Pharmacodynamic Predictors of Clinical Potency for Hepatitis C Virus Nonnucleoside Polymerase and Protease Inhibitors. *Antimicrob. Agents Chemother.* **56**, 3144–3156 (2012).

5. Duval, X. *et al.* Amprenavir Inhibitory Quotient and Virological Response in Human Immunodeficiency Virus-Infected Patients on an Amprenavir-Containing Salvage Regimen without or with Ritonavir. *Antimicrob. Agents Chemother.* **46**, 570–574 (2002).
6. Shen, L. *et al.* Dose-response curve slope sets class-specific limits on inhibitory potential of anti-HIV drugs. *Nat. Med.* **14**, 762–766 (2008).
7. Shen, L. *et al.* A Critical Subset Model Provides a Conceptual Basis for the High Antiviral Activity of Major HIV Drugs. *Sci. Transl. Med.* **3**, 91ra63-91ra63 (2011).

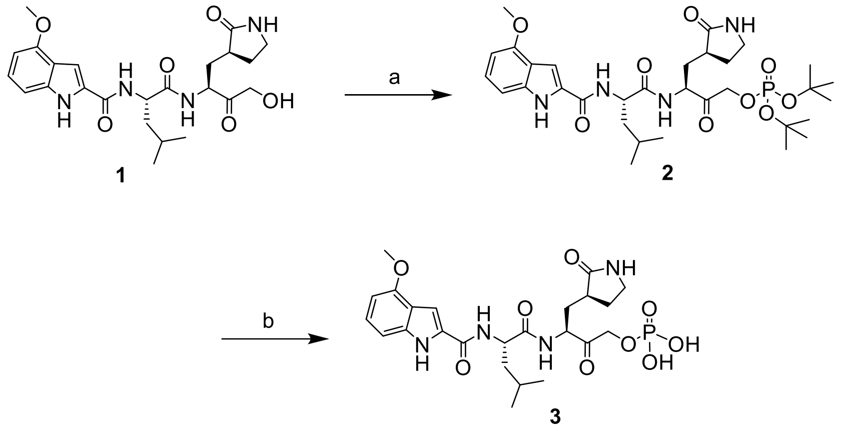

Fig. S1. Synthesis of PF-07304814 and PF-00835231 with reagents and conditions. **a**) Di-tert-butyl N,N-dipropan-2-ylphosphoramidite, tetrazole, tetrahydrofuran, 0°C to room temp., then H2O2, 0°C, 91% over 2 steps; **b**) CF3COOH, CH2Cl2, 0°C, 54%.

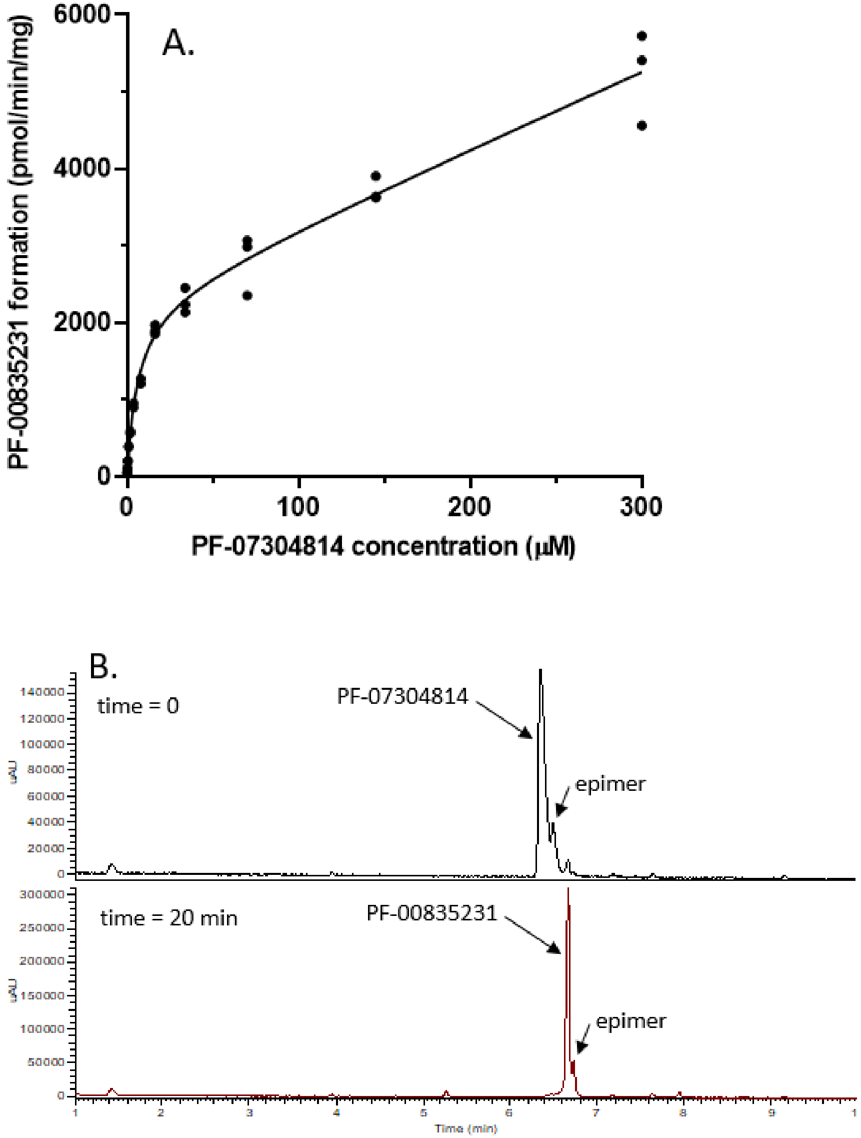

Fig. S2. Metabolism of PF-07304814 in human liver S9 (**A)** Substrate saturation plot of the metabolism of phosphate prodrug PF-07304814 to the active drug PF-00835231 in human liver S9 fraction. (**B**) HPLC-UV chromatograms of extracts of an incubation of phosphate prodrug PF-07304814 (Rt 6.3 min) in human liver S9 demonstrating complete conversion to the active entity PF-00835231 (Rt 6.7 min).

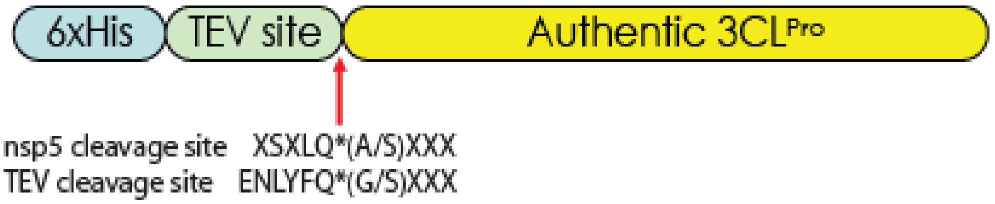

Fig. S3. 3CL protease expression constructs and associated TEV and nsp5 cleavage sites.

Table S1. Summary of the In Vitro Antiviral Activity for PF-00835231 on Related Coronaviruses in Vero E6 cells with Efflux Inhibitor

Drug Treatment	EC_{50} SARS-CoV-2 µM ± Std. Dev	EC_{50} MERS µM ± Std. Dev	EC_{50} SARS-CoV µM ± Std. Dev	EC_{50} MA-15 µM ± Std. Dev
PF-00835231 + 2 µM EI	0.06 ± 0.03	0.04 ± 0.01	0.09± 0.04	0.08 ± 0.01

EI = efflux inhibitor (CP-00100356); Std. Dev. = standard deviation; N=3 for SARS-CoV-2 and MERS: N=2 for SARS-CoV and MA-15

Table S2. Activity of PF-00835231 against human proteases and HIV protease.

Protease	IC_{50} µM
SAR-Cov-2 $3CL^{pro}$	0.0069
Human Cathepsin B	6.1
Human Elastase	>33
Human Chymotrypsin	>100
Human Thrombin	>100
Human Caspase 2	>33
Human Cathepsin D	>11
HIV-1 protease	>11

Table S3. Summary of the In Vitro Antiviral Activity, Cytotoxicity, and Therapeutic Index for PF-00835231 with and without P-gp Inhibitor

Virus Strain[a]	Host Cell	Compound	P-gp Inhibitor[b] (µM)	EC_{50} (µM)	CC_{50} (µM)	TI[c]
SARS-CoV2	Vero	CP-100356[d]	-	23.4	29.5	1.26
SARS-CoV2	Vero	PF-00835231[d]	0	35.9	>50	1.39
SARS-CoV2	Vero	PF-00835231[e]	0.5	2.36	>50	21.2
SARS-CoV2	Vero	PF-00835231[e]	1	0.95	>50	52.6
SARS-CoV2	Vero	PF-00835231[d]	2	0.46	>50	109

a. Data generated at Southern Research Institute (SRI) – 2020. SARS-CoV-2 Washington strain.
b. P-gp Inhibitor is CP-100356.
c. The TI was calculated by dividing the individual CC_{50} by the EC_{50} values and then calculating TI mean.
d. Values are averages based on an n=2 (EC_{50}) and n=1 (CC_{50}).
e. Values are the geometric means based on n=4 (EC_{50}) and n=2 (CC_{50}).

Table S4. Evaluation of the fractional metabolism of PF-00835231 in human liver microsomes and recombinant CYP3A using selective CYP3A inhibitor ketoconazole.

Metabolite	K_M (µM)	V_{max} (pmol/min/mg)	CL_{int} (µL/min/mg)	f_{CL}	% Inhibition by ketoconazole	f_m (CYP3A)
Metabolite 1	124	321	2.6	0.61	86	0.53
Metabolite 2	119	91	0.76	0.18	90	0.16
Metabolite 3	120	68	0.57	0.13	82	0.11
Metabolite 4	43	12	0.30	0.07	82	0.06
**Sum**			**4.2**			**0.86**

CL_{int} = Intrinsic clearance; CYP = Cytochrome P450; f_{CL} = Fractional clearance; f_m = Fraction metabolized; HLM = Human liver microsomes; K_M = Concentration at 50% maximum velocity; rCYP = Recombinant human CYP; V_{max} = Maximum initial velocity.

Table S5. Reversible CYP inhibition by PF-07304814 or PF-00835231 in human liver microsomes using individual CYP substrates in the presence of NADPH.

CYP	Enzyme Reaction	Zero-Minute Preincubation (T_0)[a]		30-Minute Preincubation (T_{30})[a]	
		PF-07304814	PF-00835231	PF-07304814	PF-00835231
		IC_{50} (μM)	IC_{50} (μM)	IC_{50} (μM)	IC_{50} (μM)
1A2	Phenacetin O-dealkylation	>100	>200	>100	>200
2B6	Bupropion hydroxylation	ND	>200	ND	>200
2C8	Amodiaquine N-dealkylation	>100	>200	>100	>200
2C9	Diclofenac 4′-hydroxylation	>100	>200	>100	>200
2C19	S-Mephenytoin 4′-hydroxylation	>100	>200	>100	>200
2D6	Dextromethorphan O-demethylation	>100	>200	>100	>200
3A4/5	Midazolam 1′-hydroxylation	>100	>200	>100	>200
3A4/5	Testosterone 6β-hydroxylation	ND	108 (70-172)	ND	62.4 (41-98)

Confidence interval shown in parenthesis; CYP = Cytochrome P450; IC_{50} = 50% inhibitory concentration; NADPH = Reduced form of nicotinamide adenine dinucleotide phosphate; NC = Not calculated; ND = Not determined, TDI = Time-dependent inhibition; T_0 = Time zero; T_{30} = Time 30 minutes.
[a]Average data obtained from triplicate samples for each test article concentrations were used to calculate IC_{50} values.

Table S6. Time dependent CYP3A4/5 inhibition by PF-00835231 in human liver microsomes using individual CYP substrates.

CYP	Probe Substrate (Concentration)	k_{inact} ± SE (min^{-1})	K_I ± SE (µM)	k_{inact} / K_I (mL/µmol/min)
3A4/5	Midazolam (20 µM)	0.0300 ± 0.0020	163 ± 31	0.184
3A4/5	Testosterone (386 µM)	0.0434 ± 0.0007	168 ± 8	0.258

CYP = Cytochrome P450; HLM = Human liver microsomes; K_I = Apparent inactivation constant at half-maximal rate of inactivation; k_{inact} = Maximal rate of enzyme inactivation; k_{inact}/K_I = Measure of inactivator efficiency; NADPH = Reduced form of nicotinamide adenine dinucleotide phosphate; SE = Standard error; TDI = Time-dependent inhibition.

Table S7. ***In vitro*** **transporter inhibition by PF-07304814 or PF-00835231 using probe substrates.**

Transporter	Probe Substrate (Concentration)	IC_{50} (µM)	
		PF-07304814	PF-00835231
MDR1/P-gp	N-methyl quinidine (0.2 µM)	>300	65.6
BCRP	Rosuvastatin (0.2 µM)	238	19.5
OATP1B1	Rosuvastatin (0.5 µM)	134	30.1
OATP1B3	Rosuvastatin (0.5 µM)	202	51.6
OCT1	[^{14}C]Metformin (10 µM)	>300	36.3
OAT1	[^{3}H]PAH (0.5 µM)	>300	>300
OAT3	[^{3}H]ES (0.1 µM)	>300	>300
OCT2	[^{14}C]Metformin (20 µM)	>300	>300
MATE1	[^{14}C]Metformin (20 µM)	>300	175.1
MATE2K	[^{14}C]Metformin (20 µM)	>300	179.8

BCRP = Breast cancer resistance protein; BSEP = Bile salt export pump; OAT = Organic anion transporter; OATP = Organic anion-transporting polypeptide; OCT = Organic cation transporter; IC_{50} = 50% inhibitory concentration; MATE = Multidrug and toxin extrusion protein; MDR = Multidrug resistance protein; PAH = P-aminohippuric acid; P-gp = P-glycoprotein.

Table S8. Plasma protein binding of PF-07304814 or PF-00835231 in plasma, liver microsomes and S9.

Species	Matrix	Unbound fraction fu (%CV)	
		**PF-07304814[d]**	**PF-00835231**
Human	Liver microsomes[a,c]	ND	0.75 (8.7)
Human	Liver S9[b,c]	0.890 (1.9)	ND
Human	Plasma	0.184 (5.9)	0.449 (7.5)
Monkey	Plasma	0.361 (10.2)	0.441 (7.7)
Dog	Plasma	0.312 (10.9)	0.416 (9.5)
Rat	Plasma	0.379[f] (12.7)	0.327[g] (12.1)

[a]measured at 0.8mg/mL protein, [b]measured at 0.03mg/mL protein, [c]2 µM substrate concentration, [d]3.4 µM substrate concentration, [e]5 µM substrate concentration, [f]Sprague-Dawley rat plasma, [g]Wistar Hannover rat plasma

Table S9. Preclinical plasma PK summary of PF-07304814 or PF-00835231 following oral and/or IV administration to rats, dogs and monkeys.

Rat PK Data following IV or oral administration		
**PK parameter**	**PF-07304814 (n=2)**	**PF-00835231 (n=3)**
Dose	1.17mg/kg	2mg/kg (IV + PO)
CL (mL/min/kg)	194 (168-220)	27.0 ± 3.10
Vdss (L/kg)	0.58 (0.57-0.59)	0.75 ± 0.24
Terminal T1/2 (h)	0.30 (0.36-0.23)	0.72 ± 0.12
Oral F%	ND	1.4 ± 0.76
%unchanged in urine	<0.1 (<0.1-<0.1)	7.8 ± 11.5
PF-00835231 AUCinf (ng.h/mL)	424 (366-481)	1250 ± 146
%Conversion to PF-00835231	68% (59-77)	-
**Dog PK Data following IV administration (n=2)**		
**PK parameter**	**PF-07304814**	**PF-00835231**
Dose (mg/kg)	1.17mg/kg	1.0mg/kg (IV)
CLp (mL/min/kg)	517 (301-733)	18.2 (15.9-20.5)
Vdss (L/kg)	9.3 (5.8-12.9)	1.1 (0.9-1.2)
Terminal T1/2 (h)	0.5 (0.3-0.7)	1.5 (1.4-1.6)
%unchanged in urine	<0.1 (<0.1-<0.1)	4.8 (1.6-8.0)
PF-00835231 AUCinf (ng.h/mL)	753 (633-872)	932 (813-1050)
%Conversion to PF-00835231	81% (78-83)	-
**Monkey PK Data following IV or oral administration (n=2)**		
**PK parameter**	**PF-07304814**	**PF-00835231**
Dose (mg/kg)	1.17mg/kg	1.0mg/kg (IV), 5mg/kg PO
Cl (mL/min/kg)	191 (129-252)	28.7 (27.1-30.2)
Vdss (L/kg)	1.8 (0.91-2.6)	1.4 (1.3-1.5)
Terminal T1/2 (h)	2.6 (2.2-3.0)	1.2 (1.1-1.3)
Oral F%	ND	<0.1 (<0.1-<0.1)
%unchanged in urine	<0.1 (<0.1-<0.1)	0.9 (0.8-1.0)
PF-00835231 AUCinf (ng.h/mL)	447 (304-589)	583 (614-552)
%Conversion to PF-00835231	76% (55-96)	-

n=2 range shown in parenthesis, n=3 ± SD shown in parenthesis, ND = not determined

Table S10. Predicted pharmacokinetic parameters of PF00835231 with and without coadministration of itraconazole.

PF-07304814[a]	Itraconazole	C_{max}		AUC		$C_{max}R$	AUCR
mg/day	mg/day	µM total	µM unbound	µM·h total	µM·h unbound	ratio	ratio
500	0	1.1	0.50	27	12	-	-
	200	2.4	1.1	58	26	2.2 (2.1-2.3)	2.1 (2.0-2.2)

Data are expressed as geometric mean for C_{max} and AUC and geometric mean with 90% confidence intervals in parentheses for $C_{max}R$ and AUCR.
[a] Daily dose of PF-07304814 provides formation of PF00835231 equivalent to 320mg/day used in the simulation.

Table S11. Metabolic stability and enzyme kinetics of PF-07304814 in liver S9.

Kinetic Parameters	Kinetics			
	Rat	Dog	Monkey	Human
Model	Two Enzyme – MM and Unsaturable	Two Enzyme – MM and Unsaturable	Two Enzyme – MM and Unsaturable	Two Enzyme – MM and Unsaturable
K_M (µM)	53.4	2.91	8.10	6.23
V_{max} (pmol/min/mg)	2188	214	1186	2313
$CL_{int,2}$ (µL/min/mg)	4.56	1.69	3.06	9.96
$CL_{int,app}$ (µL/min/mg)	45.5	75.2	150	381
CL_{intu} (µL/min/mg)	51	84	168	428

$CL_{int,app}$ = Apparent intrinsic clearance; $CL_{int,2}$ = Apparent intrinsic clearance of low affinity kinetic component; CL_{intu} = unbound intrinsic clearance calculated by CLint/ fu inc. K_M = Michaelis-Menten constant; MM = Michaelis-Menten; NADPH = β-Nicotinamide Adenine Dinucleotide Phosphate; S9 = Subcellular fraction; V_{max} = Maximum enzyme velocity.

Table S12. Physicochemical and pharmacokinetic parameters of PF00835231 for PBPK modeling.

Parameter (units)	Value	Source
Molecular weight	472	Calculated
LogP	0.75	Calculated
pK_a	neutral	Calculated
Rbp	0.8	Measured *in vitro*
$f_{u,plasma}$	0.449	Measured *in vitro*
V_{ss} (L/kg)	1	Predicted from animal data
Kp scalar	3.1	Adjusted from the prediction (method 2)
CL_{plasma} (L/h)	25	Predicted from *in vitro* CL
$f_{m,CYP3A}$	0.76	Predicted from *in vitro* phenotyping data
$rCL_{int,CYP3A4}$ (µL/min/pmol P450)	0.15	Calculated by Simcyp (retrograde model)
$CL_{int,HLM}$ (µL/min/mg protein)	6	Calculated by Simcyp (retrograde model)
CL_{renal} (L/h)	3	Predicted from animal data
CYP3A4 K_i (µM)	108	Measured *in vitro*
CYP3A4 K_I (µM)	163	Measured *in vitro*
CYP3A4 k_{inact} (h^{-1})	1.8	Measured *in vitro*
