# Supplementary material for: Preclinical characterization of an intravenous coronavirus 3CL protease inhibitor for the potential treatment of COVID19
Source: Nat Commun. 2021 Oct 18;12:6055. doi: 10.1038/s41467-021-26239-2 (PMC8523698; doi:10.1038/s41467-021-26239-2)
Supplement: Supplementary file 3 — Reporting Summary [file 41467_2021_26239_MOESM3_ESM.pdf]

nature research

Corresponding author(s): Rhys Jones

Last updated by author(s): 08-30-2021

Reporting Summary

Nature Research wishes to improve the reproducibility of the work that we publish. This form provides structure for consistency and transparency in reporting. For further information on Nature Research policies, see our Editorial Policies and the Editorial Policy Checklist.

Statistics

For all statistical analyses, confirm that the following items are present in the figure legend, table legend, main text, or Methods section.

n/a	Confirmed	
☐	☒	The exact sample size (*n*) for each experimental group/condition, given as a discrete number and unit of measurement
☐	☒	A statement on whether measurements were taken from distinct samples or whether the same sample was measured repeatedly
☐	☒	The statistical test(s) used AND whether they are one- or two-sided<br>*Only common tests should be described solely by name; describe more complex techniques in the Methods section.*
☒	☐	A description of all covariates tested
☐	☒	A description of any assumptions or corrections, such as tests of normality and adjustment for multiple comparisons
☐	☒	A full description of the statistical parameters including central tendency (e.g. means) or other basic estimates (e.g. regression coefficient) AND variation (e.g. standard deviation) or associated estimates of uncertainty (e.g. confidence intervals)
☐	☒	For null hypothesis testing, the test statistic (e.g. *F*, *t*, *r*) with confidence intervals, effect sizes, degrees of freedom and *P* value noted<br>*Give P values as exact values whenever suitable.*
☒	☐	For Bayesian analysis, information on the choice of priors and Markov chain Monte Carlo settings
☒	☐	For hierarchical and complex designs, identification of the appropriate level for tests and full reporting of outcomes
☒	☐	Estimates of effect sizes (e.g. Cohen's *d*, Pearson's *r*), indicating how they were calculated

Our web collection on statistics for biologists contains articles on many of the points above.

Software and code

Policy information about availability of computer code

Data collection	No software was used.
Data analysis	The melting temperature (Tm) was calculated as the mid-log of the transition phase from the native to the denatured protein using a Boltzmann model in Protein Thermal Shift Software v1.3. In vitro IC50 values: IC50 values were generated based on a four-parameter logistic fit model using ActivityBase software (IDBS). In vitro Single agent studies: For the image analysis, the SpotDetector bio-application from the Cellomics software was used, which requires two (2) channels, one (1) for object identification (first channel) and one (1) for signal/spots identification (second channel). The Valid Object Count reported value was the total count of the number of nuclei. In vitro Combination studies: Images were analyzed using the Multi-Wavelength Cell Scoring Application Module (MetaXpress). GeneData Screener was used to assess drug combination effects, at 90% effect, using the Loewe, Bliss, and HSA models or SynergyFinder using the ZIP model. Sars-Cov2 mouse animal model: The Prism software (GraphPad) was used to determine differences in lung titers using T tests on log transformed data. ADME studies: Analytes were quantified versus a standard curve using GraphPad Prism v8 (San Diego, CA) or Sciex Analyst software. The slope (-kobs, observed rate) of each line was then calculated for the linear portion of the curve and the non linear outliers, as determined by GraphPad automatic outlier elimination, were excluded. The half-maximal inhibitory concentration (IC50) for each transporter was calculated in GraphPad Prism. Pharmacokinetic parameters were calculated using noncompartmental analysis (Watson v.7.5, Thermo Scientific). PBPK simulations: A commercially available dynamic PBPK model, Simcyp population-based simulator (version 18.2; Certara UK Limited, Simcyp Division, Sheffield, United Kingdom), was used in the present study. GraphPad (v9.0.0) was used for statistical analysis

For manuscripts utilizing custom algorithms or software that are central to the research but not yet described in published literature, software must be made available to editors and reviewers. We strongly encourage code deposition in a community repository (e.g. GitHub). See the Nature Research guidelines for submitting code & software for further information.

Data

Policy information about availability of data

All manuscripts must include a data availability statement. This statement should provide the following information, where applicable:

- Accession codes, unique identifiers, or web links for publicly available datasets
- A list of figures that have associated raw data
- A description of any restrictions on data availability

The data for both the main and supplemental figures has been uploaded to www.Figshare.com. The links to given in the data availability section of the manuscript.

Field-specific reporting

Please select the one below that is the best fit for your research. If you are not sure, read the appropriate sections before making your selection.

[x] Life sciences   [ ] Behavioural & social sciences   [ ] Ecological, evolutionary & environmental sciences

For a reference copy of the document with all sections, see nature.com/documents/nr-reporting-summary-flat.pdf

Life sciences study design

All studies must disclose on these points even when the disclosure is negative.

Sample size	5 mice per group in the Sars-CoV study, 6 mice per group is Sars-CoV-2 mouse study. Rat PK studies were n=2-3 per group, Dog PK studies were n=2 per group, Non-human primate studies were n=2 per group. GLP toxicity studies were 15 Sprague-Dawley rats per sex. Non-GLP studies were 3 rats per group.
Data exclusions	No data was excluded
Replication	in vitro data was replicated 3-13 times as described in figure 2.<br>Combination efficacy was replicated 3 times<br>Thermal shift was replicated 10 times as described in the paper.
Randomization	Mice in efficacy studies were randomly assigned to a treatment group. Rats in toxicity and PK studies were randomly assigned to treatment groups. Dogs and Monkeys in PK studies were randomly assigned to treatment groups
Blinding	Investigators (data collection and analysis) were blinded to compound identity.

Reporting for specific materials, systems and methods

We require information from authors about some types of materials, experimental systems and methods used in many studies. Here, indicate whether each material, system or method listed is relevant to your study. If you are not sure if a list item applies to your research, read the appropriate section before selecting a response.

Materials & experimental systems

n/a	Involved in the study	
[ ]	[x]	Antibodies
[ ]	[x]	Eukaryotic cell lines
[x]	[ ]	Palaeontology and archaeology
[ ]	[x]	Animals and other organisms
[x]	[ ]	Human research participants
[x]	[ ]	Clinical data
[x]	[ ]	Dual use research of concern

Methods

n/a	Involved in the study	
[x]	[ ]	ChIP-seq
[x]	[ ]	Flow cytometry
[x]	[ ]	MRI-based neuroimaging

Antibodies

Antibodies used	Rabbit polyclonal SARS Nucleocapsid Protein Antibody [NB100-56576] (Novus Biologicals, Centennial, CO), 1:400. The combo in vitro studies were polyclonal patient sera from 2 recovered patients. The Goat anti-Human IgG (H+L) Cross-Adsorbed Secondary Antibody, Alexa Fluor 488 was purchased from Invitrogen (cat # A-11013).
Validation	The rabbit polyclonal SARS antibody and the Goat anti-Human IgG antibody used are commercially available have been validated by the manufacturer. The polyclonal patient sera antibodies were validated and used for compound screening as published in: Garcia, G. et al. Antiviral drug screen identifies DNA-damage response inhibitor as potent blocker of SARS-CoV-2 replication. Cell Rep. 35, (2021). Human polyclonal sera from COVID-19 patients were screened for uninfected cell staining and other off target staining. It was confirmed that the sera specifically stained SARS-CoV-2 infected cells.

Eukaryotic cell lines

Policy information about cell lines

Cell line source(s)	VeroE6 Cells - American Type Culture Collection<br>MRC-5 Cells- American Type Culture Collection<br>HeLa-ACE2- stable cell line (Deli Huang, The Scripps research institute)<br>HEK293-MATE1 and HEK293-MATE2K- Dr. Katsuhisa Inoue (Nagoya City University)<br>HEK293-OAT1 and HEK293-OAT3 Cells Dr. Kathleen Giacomini (UCSF, California)<br>HEK293-OATP1B1 Cells Absorption Systems (Exton, Pennsylvania)<br>HEK293-OATP1B3 Cells Pfizer (Sandwich, United Kingdom)<br>HEK293-OCT1 and HEK293-OCT2 Cells Dr. Kathleen Giacomini (UCSF, California)
Authentication	Cell lines that were obtained from vendors were authenticated by those vendors or scientific collaborators
Mycoplasma contamination	MRC-5 cells were tested for contamination and were negative by the MycoAlert Mycoplasma Detection kit (Lonza-LT07-318).<br>HeLa cells tested for contamination and were negative by the MycoAlert Mycoplasma Detection kit.<br>For the HEK293 cells: Cells were cultured using media with gentamicin. All products such as media are for cell culture grade (sterile).<br>VeroE6 cells were deterined to be mycoplasma free by PCR analysis. Aliquots of those cells were dispensed into cryovials, frozen and stored under liquid nitrogen. Cells for the assay were propagated from the frozen stock. A cell batch undergoes fewer than thirty passages before they are discarded and a new batch propagated from the frozen stock.
Commonly misidentified lines (See ICLAC register)	None

Animals and other organisms

Policy information about studies involving animals; ARRIVE guidelines recommended for reporting animal research

Laboratory animals	Wistar-Hannover Rats – Charles River laboratories or Vital River and were typically 7-10 weeks of age at the time of dosing<br>Male Beagle dogs were purchased from Marshall BioResources (North Rose, New York) and were typically 1-5 years of age at the time of dosing.<br>Male Cynomolgus monkeys were purchased from Covance (Princeton, NJ), Charles River Laboratories, Inc. (Wilmington, MA), or Envigo Global Services (Indianapolis, IN); animals 3-8 years of age were used in PK studies.<br>SARS-CoV-2 Female 8-week-old specific pathogen–free BALB/c mice (the Jackson laboratory strain 000651)<br>SARS-CoV Female 8-10 week-old BALB/c mice (Charles River Cat. No. 028)<br>Non-GLP tox: Rat Sprague-Dawley male 8 week old (Charles River)<br>GLP tox: Rat Sprague-Dawley male and female 8 week old (Charles River)
Wild animals	No wild animals were used
Field-collected samples	none
Ethics oversight	SARS-CoV - Animal treatment and subsequent observations of animal weight, and virus titration were conducted in the BSL-3 laboratory at University of Maryland College Park under IACUC approved protocols.<br>SARS-CoV-2- Animal studies using the Ad5-hACE2 mouse model were performed in animal biosafety level 3 (BSL3) facility at the Icahn school of Medicine in Mount Sinai Hospital, New York City. All work was conducted under protocols approved by the Institutional Animal Care and Use Committee (IACUC).<br>Animal PK studies (rat, dog, and non-human primate)- All activities involving animals were carried out in accordance with federal, state, local and institutional guidelines governing the use of laboratory animals in research in an AAALAC accredited facility and were reviewed and approved by Pfizer's Institutional Animal Care and Use Committee.<br>Animal tox studies- All procedures performed on the animals were in accordance with regulations and established guidelines and were reviewed and approved by Pfizer's Institutional Animal Care and Use Committee.

Note that full information on the approval of the study protocol must also be provided in the manuscript.
